# The most relevant diagnostic criteria for developmental dysplasia of the hip: a study of British specialists

**DOI:** 10.1186/s12891-016-0867-4

**Published:** 2016-01-19

**Authors:** Daniel Williams, Evangelia Protopapa, Kuldeep Stohr, James B. Hunter, Andreas Roposch

**Affiliations:** Department of Orthopaedic Surgery, Great Ormond Street Hospital for Children, Great Ormond Street, London, WC1N 3JH UK; Institute of Child Health, University College, London, UK; Addenbrooke’s Hospital, Cambridge, UK; Nottingham University Hospital, Nottingham, UK

**Keywords:** Developmental, Dysplasia, Hip, Diagnosis

## Abstract

**Background:**

Developmental dysplasia of the hip (DDH) is the most common orthopaedic disorder in newborns. Despite this considerable variation in practice exists. The aim of this study was to determine the clinical relevance and a ranking order for the diagnostic criteria in DDH amongst paediatric orthopaedic surgeons practicing in the UK.

**Method:**

One hundred members of the British Society of Children’s Orthopaedic Surgery (BSCOS) were asked to rate the importance of 37 criteria useful in the diagnosis of DDH in newborns, using a 10 cm visual analogue scale. We determined the consistency among specialists in rating the criteria with the intraclass correlation coefficient (ICC) and compared the results to a group of international peers.

**Results:**

Ortolani/Barlow tests, asymmetry in abduction ≥20° and a first-degree relative treated for DDH ranked among the top ten. Participants demonstrated poor consistency in rating the 37 criteria (ICC 0.39; 95 % CI 0.29, 0.52), but for clinical examination criteria alone their consistency improved (ICC 0.52; 0.35, 0.75). The importance ratings of members of BSCOS and members of the European Paediatric Orthopaedic Society differed for 15/37 (41 %) criteria (p <0.05).

**Conclusions:**

Members of BSCOS had a preference for criteria relating to clinical examination and ultrasound.

**Electronic supplementary material:**

The online version of this article (doi:10.1186/s12891-016-0867-4) contains supplementary material, which is available to authorized users.

## Background

Early recognition of developmental dysplasia of the hip (DDH) associated with better outcomes [1]. Clearly defined, well formulated diagnostic criteria are vital to identify infants needing observation or treatment. This is particularly important during the first 8 weeks of life when there is perhaps greatest uncertainty as to the capacity for spontaneous resolution of abnormal findings of the hip [2–4]. In an attempt to elicit clearly defined, well-formulated diagnostic criteria for DDH in this age group, a Delphi consensus study of paediatric orthopaedic surgeons from 34 countries was conducted [5]. It identified 37 standardized diagnostic criteria for DDH in this age group. Details of criteria are shown in Additional file 1. In the present study we sought to discern the opinions of British paediatric orthopaedic surgeons on these 37 criteria. Specifically, we wanted to determine (1) a ranking order of clinical relevance of these criteria reflecting the opinions of surgeons practicing in the UK, (2) the consistency with which British paediatric orthopaedic surgeons agree about the importance of these criteria and (3) how their opinions compare to a group of international paediatric orthopaedic surgeons [6].

## Methods

The study was approved by the institutional review board for Great Ormond Street Hospital and the Institute of Child Health, British Society of Children’s Orthopaedic Surgery (BSCOS) and European Paediatric Orthopaedic Society (EPOS). We surveyed members of BSCOS and presented them with a set of 37 criteria grouped in 4 domains; patient history, clinical examination, ultrasonography, radiography. These had been compiled in an international consensus study [5]. We asked survey participants to rate each criterion on a 10-cm visual analogue scale (VAS) for its relative importance in making the diagnosis of DDH in infants not older than 8 weeks. We defined DDH as a condition requiring either treatment or follow-up with an orthopaedic surgeon. We employed Dillman’s tailored design method for survey design and conduct [7]; it entails making up to 4 contacts with participants by first-class mail or e-mail, personalized correspondence and additional contacts by telephone or fax.

We surveyed 148 eligible members of BSCOS. All surgeons in this study were specialists who examine and treat infants for DDH as part of their routine practice. We were solely interested in the surgeons’ opinion on each criterion in isolation, rather than in determining how surgeons establish the diagnosis of DDH using combinations of these criteria. As such, we asked the participants to rate each of the 37 criteria irrespective of any other abnormalities. We recognize that this may not reflect how clinicians arrive at a diagnosis; however, because each criterion was rated in isolation we assumed that the relative importance rating would be stable [8]. We compiled the VAS means (a ratio scale measurement of the perceived value on the VAS provided a continuous outcome) for all criteria based on the responses of all members of BSCOS. Based on these VAS means we generated a ranking list and defined the top ten criteria. We compared VAS means to those of members of EPOS using the signed rank test at the 5 % significance level.

We determined the consistency of members of BSCOS in assigning the importance rating to each criterion with the ICC. The concept of consistency is defined as the agreement of two quantitative measurements in settings where neither one is assumed correct [9]. Multiple raters evaluated all of the criteria and the case 2 model according to Shrout and Fleiss [10] was employed. ICC is interpreted as follows: ≤0.40, poor consistency or large variation in opinion; 0.41 to 0.74, acceptable consistency; and ≥0.75 good consistency [11]. A sample size of 37 items with 148 raters for each criterion achieves 80 % power to detect an ICC of 0.80 under the alternative hypothesis when the ICC under the null hypothesis is 0.69, using an F-test and a 5 % significance level [12].

## Results

68 % (100/148) members of BSCOS responded to the survey. BSCOS members expressed a preference for clinical examination criteria, which constituted 6 of the top ten raking criteria (Table 1). They included Ortolani and Barlow tests, asymmetry in abduction ≥20° and leg length discrepancy. Among the top ten ranked 3 ultrasonographic criteria: sonographically dislocatable hip, α angle <45°, and femoral head displacement >2 mm from the medial aspect of the acetabulum. A first degree relative treated for DDH was the only risk factor ranking in the top ten. The details for the ranking of each criteria by BSCOS members are shown in Table 2.

**Table 1:** Top ten ranking criteria based on group means of BSCOS and EPOS

Rank	BSCOS	EPOS
1	Ortolani test positive	Ortolani test positive
2	Barlow test positive	Barlow test positive
3	Asymmetry in abduction ≥20°	Dislocatable hip on dynamic ultrasound
4	Dislocatable hip on dynamic ultrasound	Asymmetry in abduction ≥20°
5	Abduction limited to 45°	α angle <45°
6	Leg-length discrepancy/Galeazzi	Femoral head displaced on stress ultrasound
7	Any asymmetry of hip abduction	Any asymmetry of hip abduction
8	α angle <45°	Breech presentation
9	Femoral head displaced on stress ultrasound	Abduction limited to 45°
10	First degree relative treated for DDH	Leg-length discrepancy/Galeazzi

**Table 2:** Ranking of criteria based on group means of the BSCOS members. Shown are means with standard deviations in parentheses

Criterion	Mean (SD)
*Physical Examination*	
Ortolani test positive	9.1 (1.1)
Barlow test positive	8.6 (1.8)
Asymmetry in abduction ≥20°	8.4 (1.7)
Abduction limited to ≤45°	7.9 (2.0)
Leg-length discrepancy/Galeazzi sign	7.9 (2.7)
Any asymmetry of hip abduction	7.8 (2.1)
Abduction limited to ≤60°	6.4 (2.5)
Torticollis	5.2 (2.7)
Abduction limited to ≤70°	5.2 (2.8)
Flexible foot deformities	5.0 (2.9)
Congenital clubfoot or other fixed foot deformities	4.0 (2.8)
Asymmetry of groin or skin crease(s)	3.2 (2.8)
Hip click	2.5 (2.1)
*Ultrasound*	
Dislocatable hip (dynamic exam)	8.1 (2.5)
α angle <45° (static exam)	7.7 (3.1)
Femoral head displaced anatomically with no congruency on stress test	7.6 (3.2)
α angle <50° (static exam)	7.2 (3.0)
Femoral head coverage ≤45 % (static exam)	7.0 (2.8)
α angle <55° (static exam)	6.4 (2.8)
Femoral head coverage ≤50 % (static exam)	6.0 (2.8)
α angle <60° (static exam)	5.4 (3.0)
Displacement of femoral head >2 mm from medial aspect of acetabulum on dynamic exam	4.9 (3.2)
Femoral head coverage ≤60 % (static exam)	4.3 (3.1)
Femoral head coverage ≤70 % (static exam)	3.3 (3.1)
*Patient characteristics and history*	
First degree relative treated for DDH	7.4 (2.2)
Breech presentation	7.2 (2.3)
Breech positioning in-utero but born by vertex delivery	5.8 (2.8)
Family history of DDH	5.7 (2.6)
Oligohydramnios	4.5 (2.6)
First born baby girl	4.4 (2.9)
Female gender	4.2 (2.8)
Birth weight >4000 g (8.8 lbs)	3.4 (2.5)
Born by caesarian section	3.2 (2.6)
Multiparous mother	3.1 (2.7)
*Radiography*	
Midpoint of the femoral metaphysis lateral to Perkins line	4.6 (3.5)
Acetabular index >30° at 8 weeks	2.7 (2.8)
Acetabular index >25°at 8 weeks	2.0 (2.3)

**Fig. 1:**
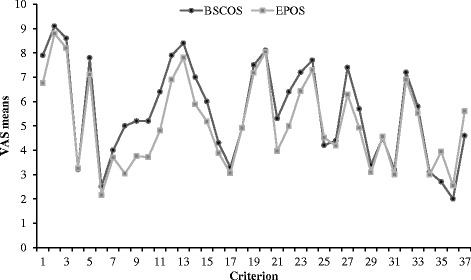
Comparison of mean ratings of EPOS and BSCOS. Details of criteria are shown in Additional file 1

VAS mean values assigned to individual criteria were largely similar between members of BSCOS and EPOS (Fig. 1). Of the 15 criteria that differed significantly, the largest differences were seen (p <0.001) for the criteria postural foot deformity, torticollis and abduction ≤70°, with members of BSCOS assigning higher mean ratings (Table 3).

**Table 3:** 15 criteria based on VAS ratings with a statistically significant difference between BSCOS and EPOS respondents. Shown are means and standard deviations

Criterion	BSCOS (*n* = 85)	EPOS (*n* = 156)	*p*-value
Leg-length discrepancy/Galeazzi sign	7.9 (2.7)	6.8 (3.0)	0.001
Any asymmetry of hip abduction	7.8 (2.1)	7.1 (2.5)	0.017
Flexible foot deformities	5.0 (2.9)	3.0 (2.5)	<0.001
Torticollis	5.2 (2.7)	3.7 (2.5)	<0.001
Abduction limited to ≤70°	5.2 (2.8)	3.7 (2.8)	<0.001
Abduction limited to ≤60°	6.4 (2.5)	4.8 (2.7)	<0.001
Abduction limited to 45°	7.9 (2.0)	6.9 (2.5)	0.001
Asymmetry in abduction ≥20°	8.4 (1.7)	7.8 (2.4)	0.019
Femoral head coverage ≤45 %	7.0 (2.8)	5.9 (3.4)	0.001
α angle <60°	5.3 (2.9)	4.0 (3.2)	0.002
α angle <55°	6.4 (2.8)	5.0 (3.2)	0.045
First degree relative treated for DDH	7.4 (2.2)	6.3 (2.6)	0.002
Any family history of DDH	5.7 (2.6)	4.9 (2.8)	0.044
Acetabular index >30° at 8 weeks	2.7 (2.8)	3.9 (3.1)	0.006
Midpoint of the femoral metaphysis lateral to Perkins line	4.6 (3.5)	5.6 (3.3)	0.047

Members of BSCOS demonstrated poor consistency in rating the 37 criteria, with an ICC of 0.39 (95 % CI = 0.29, 0.52). Better consistency was found for criteria relating to the clinical examination – the ICC was 0.52 (0.35, 0.75). Members of BSCOS were least consistent in their opinions about the importance of criteria relating to hip ultrasound, with an ICC of 0.25 (0.14, 0.52). Poor consistency was noted also for criteria of patient history (ICC 0.39; 0.23, 0.69) and radiography (ICC 0.31; 0.10, 0.95).

## Discussion

This study determined the opinions of British paediatric orthopaedic surgeons about a set of 37 criteria which have been identified as the most relevant features for diagnosing DDH in the first 8 weeks of life [5]. Because all 37 criteria cannot be equally important, we wanted to delineate those identified as most and least important by British specialist surgeons and determine to what degree their opinions differ compared to specialists from other countries.

We note the potential limitations of this study. As clinical experience and exposure accumulate the symptoms and signs associated with a diagnosis are “chunked” together and not taken in isolation. By asking experts to rank individual criteria we have established the opinions of surgeons and this may not reflect their normal practice. However, individual criteria are important as they can act as a trigger to activate the relevant knowledge.

Surveys are an effective means of evaluating physicians’ attitudes [13] and evidence suggests that physicians act as they indicate in surveys [14]. The response rate of this survey was 68 %, however, this is reasonable considering that the mean response rate of surveys involving physicians is 54 % [15].

Members of BSCOS rated historically well-established diagnostic criteria such as the Ortolani test highest and controversial ones such as hip click lowest despite this being a common reason for referral. The opinions of British surgeons were consistent with an international group of paediatric orthopaedic surgeons – the top ten ranking criteria were identical with the exception that breech presentation was not included in the top-ten of the BSCOS panellists. The pattern of importance ratings was almost identical between BSCOS and EPOS (Fig. 1).

The fact that criteria related to the clinical examination were among the highest ranking in this study reflects other studies from the UK about the diagnosis of DDH in early infancy. Clarke et al. [16] based triage decisions of infants not older than 3 days on Ortolani, Barlow and Galeazzi tests. Talbot et al. [17] and Price et al. [18], examining the same age group, used Ortolani and Barlow tests but placed no emphasis on the Galeazzi test. Limited hip abduction was not reported as a diagnostic criterion in either of these 3 large studies. However, limited abduction ≥20° ranked third amongst BSCOS members. In fact, 3 criteria relating to hip abduction ranked top ten in our study; asymmetry in abduction ≥20°, abduction limited to 45° and any asymmetry in abduction. In contrast, a study of infants aged 3 to 10 months highlights the lack of reliability when relying on clinical examination alone, with 46 % of infants without DDH exhibiting a limit to hip abduction [19]. Of note, members of EPOS placed less value on the criterion hip abduction.

In terms of risk factors, members of BSCOS ranked highest family history and breech presentation (Table 2). This is in keeping with current practice: 3 recent studies on screening in DDH [16–18] utilized these 2 criteria to select at-risk patients prompting specialist referrals. In a study of 64 670 births, Talbot et al. [17] evaluated the incidence of DDH in patients with these 2 risk factors; the incidence was 3.2 % with a family history of DDH and 2.5 % with a breech presentation. Price et al. [20] examined, amongst others, the risk factors oligohydramnios and foot deformity but did not comment on their associations with DDH, suggesting that they had little value in predicting DDH. This is consistent with our survey these 2 criteria are among the lowest ranking.

While our study showed that members of BSCOS regarded ultrasound criteria as important in general, it also confirmed the ongoing controversy [2, 21] about the nature of ultrasound criteria. Three recent studies about hip screening showed that the ultrasound criteria by which surgeons defined DDH varied in the UK. While one study [16] utilized criteria based on dynamic ultrasound, another study [17] relied on the α angle in combination with dynamic criteria, and a third study [18] relied solely on the α angle. Our survey reflected this controversy – a dislocatable hip seen on dynamic ultrasound and an α angle <45° ranked among the top ten criteria, similar to the opinions of members of EPOS, but also the femoral head coverage as measured by ultrasound was rated highly (Table 2).

In quantifying how consistent members of BSCOS were in rating the 37 criteria we used the ICC. It provides a measure of the extent to which any single member identified at random would compare to any other randomly selected member. Coefficients for judgments on individual patients should reach values of 0.70 to 0.80 [22]. The best value that members of BSCOS reached was 0.52, indicating acceptable agreement about clinical examination criteria. Similar patterns were seen in an international study where paediatric orthopaedic surgeons were most consistent about clinical examination criteria [6]. In contrast, for criteria relating to patient history, ultrasound and radiography, large variations in the opinions of UK surgeons were seen. Members of BSCOS were least consistent about the ultrasonographic criteria; this may be related to the inconsistent evidence in terms of the use of this diagnostic modality. It also reflects current practice in the UK: 3 recent studies about hip screening employed different ultrasonographic criteria in defining DDH [16–18].

## Conclusion

The ranking order of criteria generated in this survey offers information for clinicians in primary and secondary care about the opinions of expert diagnosticians. Clinicians can determine how their personal preferences for diagnostic criteria differ from those experts. Such a comparison may reassure clinicians that their practice is mirrored by others, or, if not, can provide a basis for reconsidering their practice.

## Additional file

Additional file 1:
**37 diagnostic criteria for developmental dysplasia of the hip in children less than 8 weeks old.** (DOCX 106 kb)

## Abbreviations

DDH: Developmental dysplasia of the hip
BSCOS: British society of children’s orthopaedic surgery
ICC: Intraclass correlation coefficient
EPOS: European paediatric orthopaedic society
VAS: Visual analogue scale
