# Supplementary material for: The most relevant diagnostic criteria for developmental dysplasia of the hip: a study of British specialists
Source: BMC Musculoskelet Disord. 2016 Jan 19;17:38. doi: 10.1186/s12891-016-0867-4 (PMC4719727; doi:10.1186/s12891-016-0867-4)
Supplement: Additional file 1: — 37 diagnostic criteria for developmental dysplasia of the hip in children less than 8 weeks old. (DOCX 106 kb) [file 12891_2016_867_MOESM1_ESM.docx]

**Additional file 1:** 37 diagnostic criteria for developmental dysplasia of the hip in children less than 8 weeks old.

| Item | Item meaning |
| --- | --- |
| 1 | leg-length discrepancy or positive Galeazzi sign |
| 2 | positive Ortolani test/sign |
| 3 | positive Barlow test/sign |
| 4 | asymmetry of groin or skin crease(s) |
| 5 | any asymmetry of hip abduction (with both hips in flexion) |
| 6 | hip click in a stable (Ortolani and Barlow negative) hip |
| 7 | congenital clubfoot or other fixed foot deformities |
| 8 | postural (flexible) foot deformities such as metatarsus adductus/varus |
| 9 | torticollis (any form) |
| 10 | abduction of both (or one) hips limited to 70 degrees (with both hips in flexion) |
| 11 | abduction of both (or one) hips limited to 60 degrees (with both hips in flexion) |
| 12 | abduction of both (or one) hips limited to 45 degrees (with both hips in flexion) |
| 13 | asymmetry in abduction of 20 degrees or more |
| 14 | on static ultrasound, femoral head coverage 45% or less (coronal view) |
| 15 | on static ultrasound, femoral head coverage 50% or less (coronal view) |
| 16 | on static ultrasound, femoral head coverage 60% or less (coronal view) |
| 17 | on static ultrasound, femoral head coverage 70% or less (coronal view) |
| 18 | on dynamic ultrasound, displacement of femoral head >2mm from the medial aspect of the acetabulum (either coronal or transverse views) |
| 19 | femoral head displaced anatomically with no congruency on stress ultrasound (either coronal or transverse views) |
| 20 | dislocatable hip on dynamic ultrasound (either coronal or axial views) |
| 21 | on static ultrasound, alpha angle of less than 60° (Graf method) |
| 22 | on static ultrasound, alpha angle of less than 55° (Graf method) |
| 23 | on static ultrasound, alpha angle of less than 50° (Graf method) |
| 24 | on static ultrasound, alpha angle of less than 45° (Graf method) |
| 25 | female gender |
| 26 | first born baby girl |
| 27 | first degree relative was treated in any way for DDH (as per parental report) |
| 28 | any family history (also non-first degree relatives) of hip dysplasia in childhood (as per parental report) |
| 29 | birth weight >4000 g (8.8 lbs) |
| 30 | Oligohydramnios (low volume of amniotic fluid on prenatal ultrasound [as per practitioner's definition] in the absence of intrauterine growth restriction, fetal anomaly or significant maternal comorbidity) during any stage of pregnancy |
| 31 | born by caesarian section |
| 32 | breech presentation born either by vaginal delivery or caesarian section |
| 33 | A history of breech positioning in-utero but born by vertex delivery |
| 34 | multiple births or pregnancies of mother (multiparous mother) |
| 35 | Acetabular Index >30 degrees at 8 weeks as a primary imaging modality in this age group |
| 36 | Acetabular Index >25 degrees at 8 weeks as a primary imaging modality in this age group |
| 37 | migration of the midpoint of the femoral metaphysis lateral to Perkins line as a primary modality in this age group (Perkins line is a vertical line drawn through the most superior lateral margin of the ossified acetabulum) |
